# Acute psilocin increased cortical activity in rat

**DOI:** 10.3389/fnins.2026.1593703

**Published:** 2026-02-04

**Authors:** Junhong Liu, Yuanyuan Wang, Ke Xia, Jinfeng Wu, Danhao Zheng, Aoling Cai, Haitao Yan, Ruibin Su

**Affiliations:** 1 Stake Key Laboratory of National Security Specially Needed Medicines, Beijing, China; 2 Nanjing University of Chinese Medicine, Nanjing, China; 3 State Key Laboratory of Magnetic Resonance and Atomic and Molecular Physics, Key Laboratory of Magnetic Resonance in Biological Systems, Wuhan Center for Magnetic Resonance, Wuhan Institute of Physics and Mathematics, Innovation Academy for Precision Measurement Science and Technology, Chinese Academy of Sciences, Wuhan, Hubei, China; 4 The Affiliated Changzhou Second People's Hospital of Nanjing Medical University, Changzhou Second People's Hospital, Changzhou Medical Center, Nanjing Medical University, Changzhou, China

**Keywords:** 5-HT2A, BOLD fMRI, depression, egr1, functional connectivity, psilocin, psychedelics

## Abstract

Psilocin, a naturally occurring hallucinogenic component of magic mushrooms, exerts notable psychoactive effects in both humans and rodents. However, the underlying mechanisms remain not fully understood. Blood-oxygenation level-dependent (BOLD) functional magnetic resonance imaging (fMRI) is a valuable tool in many preclinical and clinical trials for investigating changes of brain activity and functional connectivity (FC) due to its noninvasive nature and widespread availability. However, fMRI effects of psilocin on rats have not been thoroughly explored. This study aimed to explore the impact of psilocin on rats’ brain activity by combining BOLD fMRI and immunofluorescence (IF) of EGR1, an immediate early gene (IEG) closely related to depressive symptoms. Ten minutes after psilocin hydrochloride injection (2.0 mg/kg, i.p.), elevated brain activity was detected in the frontal, temporal, and parietal cortex (including the cingulate cortex and retrosplenial cortex), hippocampus, and striatum. Moreover, a region-of-interest (ROI) -wise FC analysis matrix indicated enhanced interconnectivity of several regions, such as the cingulate cortex, dorsal striatum, prelimbic, and limbic regions. Further seed-based analyses revealed increased FC of cingulate cortex with the cortical and striatal areas. In addition to the fMRI observations, acute psilocin led to an increase in the EGR1 level in most cortical and striatal regions, indicating a consistent activation throughout the cortical and striatal areas. In conclusion, the psilocin-induced hyperactive state in rats is congruent to that in humans, and the increased brain activity, enhanced functional connectivity and up-regulation of EGR1 may be responsible for its pharmacological effects.

## 1 Introduction

Psilocin and psilocybin are naturally occurring hallucinogenic components derived from magic mushrooms. Psilocybin exerts psychoactive effects after being metabolized to psilocin, a chemical analog of the neurotransmitter serotonin (5-HT). Like other psychedelics such as lysergic acid diethylamide (LSD) and ayahuasca, psilocin can induce hallucination in humans, and also shows great therapeutic potential to several psychiatric disorders like depression and anxiety, which is primarily achieved by activation of the brain’s serotonin system (Moreno et al., 2006; Thomas et al., 2017; Preller et al., 2020; Vollenweider and Preller, 2020; Daws et al., 2022). Binding studies indicate psilocin has a high affinity for most serotonin receptors, including serotonin 5-HT_1A_ and 5-HT_2A_ receptors (NIMH Psychoactive Drug Screening Program database: https://pdsp.unc.edu/databases/pdsp.php), which are both important targets in the therapy of psychiatric disorders and are considered to constitute a bipartite model to modulate brain serotonin function (Carhart-Harris and Nutt, 2017).

Functional magnetic resonance imaging (fMRI) is becoming increasingly popular in basic research and drug development due to its non-invasive nature, good cross-species consistency, widespread availability, and unparalleled brain-wide spatial and temporal resolution, making it a representative platform for translational and reverse-translational studies across humans and rodents (Lu et al., 2012; Sumiyoshi et al., 2019). In the past few decades, with the advancements of hardware apparatus and data processing methodologies, a large number of studies have used fMRI technique to explore brain activity changes induced by psychiatric disorders and the chemical interventions (Carhart-Harris et al., 2016; Müller et al., 2018; Roseman et al., 2018; Zheng et al., 2018; Daws et al., 2022). The effects of psychedelics (such as psilocin, psilocybin, LSD and ayahuasca) on brain activity and functional connectivity (FC) in humans have been investigated from different perspectives. Among these studies, altered activity was mostly reported in such “hub regions” as the anterior cingulate cortex (ACC), posterior cingulate cortex (PCC), medial prefrontal cortex (mPFC), and thalamus (see the review for detail (McCulloch et al., 2022)). For example, increased FC between ACC and PCC was observed after psilocybin treatment in major depressive patients (Doss et al., 2021), and optogenetic activation of mice mPFC also had antidepressive effects (Covington et al., 2010). It seems these ‘hub regions’ are crucial for depression-relieving effects of psychedelics in humans and mice. Similarly, the rat cingulate cortex is fairly significant for interhemispheric communications despite some structural differences (Vogt and Paxinos, 2014). However, whether the cingulate regions in rats are crucial targets on psilocin-induced brain activity changes have not been fully elucidated. Therefore, we hypothesized that these hub regions, like the cingulate cortex, may be the key regions affected by psilocin in rats.

When it comes to those important fMRI studies in humans, limitations still exist. For example, fMRI readouts are an indirect reflection of neural mass activity, which are influenced by both physical and biological factors (Logothetis, 2008). As various methodologies have emerged, different researches may lead to differential results with the same data. Multifarious experimental designs and methodological techniques make it difficult to direct compare the results of different fMRI studies. In addition, it is not easy to directly compare the fMRI readouts with the postmortem molecular expression in human studies, while this can be easily done in animals (Sumiyoshi et al., 2019).

Unlike the indirect fMRI, immediate early genes (IEG) in the brain are critical mediators and direct indicators of neuron activities. These IEGs are involved in various neural processes such as learning, memory, neuroplasticity, neural response to various stimuli, and psychiatric disorders (González-Maeso et al., 2007; Pérez-Cadahía et al., 2011; Leal et al., 2014; Minatohara et al., 2016; Chandra and Lobo, 2017; Duclot and Kabbaj, 2017; Jefsen et al., 2021). IEG *early growth factor 1* (*egr1*), a member of the *egr* family (Beckmann and Wilce, 1997), is widely expressed throughout the brain (Knapska and Kaczmarek, 2004) and its translational product, EGR1 protein (EGR1), is evolutionarily conserved across humans, mice, and rats (Poirier et al., 2008). Importantly, EGR1 level is closely associated with depression, anxiety, and schizophrenia (Duclot and Kabbaj, 2017). EGR1 level in mPFC has been reported to be directly related to the depressive phenotypes (Covington et al., 2010), and have even been recommended as a marker for positive responses to antidepressant-like treatment (Minatohara et al., 2016). In patients with schizophrenia, decreased *egr1* mRNA levels in the dorsolateral prefrontal cortex (dlPFC) have been observed, while there was an increase in fibroblasts and whole blood samples, with the latter being associated with delusion states of schizophrenia patients (Yamada et al., 2007; Kurian et al., 2011; Cattane et al., 2015). Another study reported *egr1* as a biomarker specifically responsive to hallucinogenic 5-HT_2A_ receptor agonists compared to non-hallucinogenic ones. Psilocin, the bioactive metabolite of psilocybin, significantly increased brain *egr1* mRNA levels in normal mice but not in 5-HT_2A_ receptor knockout mice (htr2a^−/−^), suggesting the essential role of 5-HT_2A_ receptor in *egr1* induction in this context (González-Maeso et al., 2007). Therefore, EGR1 can be considered a direct and specific indicator of neural activity induced by psilocin.

Considering the noninvasive but indirect nature of fMRI and invasive but direct properties of IF in reflecting neural activities, we combined BOLD fMRI and IF of EGR1, for the first time, to further investigate the effects of psilocin on rat brain activity.

### Revision statement

This manuscript is a revised version of the original publication (Liu et al., 2023).

Following publication, a thorough review of the work and all original records was conducted. We discovered that the compound used in this study was actually Psilocin·HCI, not Psilocybin, as initially reported. This finding was confirmed through mass spectrometry and nuclear magnetic resonance techniques. Although both compounds exert their effects through the active metabolite psilocin, we have decided to retract the original manuscript and replace it with a corrected version to ensure the accuracy and integrity of the scientific record.

## 2 Materials and methods

### 2.1 Animals

A total of 91 male Sprague Dawley (SD) rats (Beijing HFK Bioscience Co. Ltd.), aged 7–9 weeks, weighing 230–260 g, were housed two or three per cage with food and water access *ad libitum*, under a 12 h:12 h light/dark cycle (light on at 7:00 a.m.), in a temperature- and humidity-controlled room (25 ± 2 °C and 60% ± 10%, respectively). They were permitted to acclimatize to the environment for a minimum of 1 week before experiments. In consideration of the enduring anesthetic effects of isoflurane on protein expression (Bunting et al., 2015) and long-term antidepressive effects of psychedelics (Thomas et al., 2017; Daws et al., 2022), each rat was used only once and 75 rats were used for head twitch response (HTR), 10 for fMRI scanning, and 6 for EGR1 IF. All experiments were carried out in accordance with the National Institute of Health Guidelines for the Care and Use of Laboratory Animals. Every effort was made to minimize discomfort to the animals.

### 2.2 Drugs and agents

Psilocin hydrochloride (Psi, synthesized by Stake Key Laboratory of National Security Specially Needed Medicines) was prepared in 0.9% sterile saline solution at concentrations of 0.2, 0.5, 1.0, and 2.0 mg/mL for the HTR experiment (Fadahunsi et al., 2022). The final dosage of 2.0 mg/kg Psi in fMRI and IF experiments was selected in reference to previous research (González-Maeso et al., 2007; Grandjean et al., 2021). For each experiment, psilocin was dissolved immediately before the experiment and stored in a dark environment.

### 2.3 Head twitch response

Before the HTR experiment, each rat was acclimatized to a new housing cage alone with fresh padding material for 20 min to alleviate manipulation- and environment-induced stress. Subsequently, rats received intraperitoneal (i.p.) administration with saline or Psi (0.2, 0.5, 1.0, 2.0 mg/kg) at a volume of 0.1 mL/100 g body weight. After that, the animals were placed back in the cage immediately and the HTR within 30 min were manually counted by three experimenters who were blind to the treated agents.

### 2.4 Immunofluorescence

Sixty minutes after treatment, the rats were deeply anaesthetized with sodium pentobarbital solution. Transcardial perfusion was then performed with 4% paraformaldehyde solution (PFA, Beijing Applygen Technologies Inc., China) and then with 0.1 M phosphate buffer (PBS) (Beijing Applygen Technologies Inc., China) for 10 min, respectively. The perfused brains were post-fixed in 4% PFA overnight and then dehydrated in 30% sucrose solution for 3–5 days. The dehydrated brain was frozen and sectioned into slices (30 μm thick) with a sliding microtome (RWD life science Co. Ltd., China) in the frontal plane and every sixth slice was selected as a series. The series was carefully washed and incubated with blocking buffer (Beyotime, China) for 2 h at 20 °C, then with anti-EGR1 primary rabbit antibody (1:1,000, Cell Signaling Technology, USA, 44D5) for 10 h. The incubated slices were washed with 0.1 M PBS followed by anti-rabbit secondary antibody (1:500, Cell Signaling Technology, USA, 4412S) immunostaining for 2 h at 20 °C. After washing, the slices were counterstained and cover slipped using DAPI-containing mounting medium (Sigma-Aldrich, USA). The immunostained slices were fluorescently imaged using a slice scanner (Olympus VS200, Japan). Finally, the IF images were processed with ImageJ (National Institute of Health, USA) in reference to the stereotaxic rat atlas (Paxinos and Watson, 2007).

### 2.5 MRI scanning

MRI experiments were conducted using a Bruker Biospec70/20USR small animal MR system (Bruker, Germany) operating at 300 MHz (7 T). A partial volume transmit coil was used for signal transmission and a 10 mm surface coil for signal reception (Bruker, Germany). Before scanning, rats were anesthetized with 4% isoflurane (RWD life science Co. Ltd., China) and underwent abdominal catheterization using a homemade drug injection device. Those animals were carefully transferred to the animal bed of the MRI scanner, and their heads were fixed with two ear bars and a tooth bar. Isoflurane was gradually reduced to 2–3% to maintain the animals in a stable state with a steady respiration rate ranging between 60 and 80 breaths per minute. In addition, a homemade hot water circulation device was employed to maintain the rats’ body temperature throughout the scanning process.

During scanning, a 3D-FLASH and a Fieldmap image was collected for head location and preparation for the phase field correction. Then, repeated fieldmap correction and tuning were performed to improve the image quality. fMRI data were collected before and 10 min after drug injection. In detail, resting-state fMRI data were acquired using gradient echo EPI (GE-EPI) sequence with the following parameters: field of view (FOV) = 21.0 × 28.0 mm^2^, matrix size = 80 × 60, repetition time (TR) = 2,000 ms, echo time (TE) = 14 ms, number of averages (NA) = 1, number of repetition (NR) = 300, spatial resolution = 0.35 × 0.35 mm^2^, slice thickness = 0.8 mm, slices number = 22 (no gap), and scan time = 10 min. A structure image was acquired at the same geometry using T2-weighted fast spin-echo sequence Turbo-RARE: FOV = 21.0 × 28.0 mm^2^, matrix size = 256 × 256, TR = 3,000 ms, TE = 12 ms, NA = 4, NR = 1, spatial resolution = 0.08 × 0.11 mm^2^, slice thickness = 0.8 mm, and slices number = 22 (no gap).

### 2.6 MRI data analysis

All fMRI data was preprocessed with afni,^1^ fsl,^2^ ANTs,^3^ and homemade MATLAB code. The MRI data was first converted to NIFITI format using <Bru2Anz converter>.^4^ Then, the image orientation and image center were corrected using <3drefit>. The tissue outside of the brain was removed using <antsRegistrationSyN> and a homemade rat brain mask. The first five volumes were removed and slicetiming was performed using <3dTshift>. Head motion correction was performed using <antsMotionCorr> and 0.01–0.1 Hz temporal filter was performed using <3dBandpass>. For spatial correction, every EPI image was first rigid-body transformed to match their own T2-weighted images, and then nonlinear transformed to match a rat standard template (Sigma Wistar Rat Brain Template (Barrière et al., 2019)) using <antsRegistrationSyN>. Finally, all the EPI images were smoothed using a FWHM of 0.3 mm^3^.

For degree centrality (DC) analysis, the correlation strengths between each voxel and every other voxel in the brain were calculated and a threshold of 0.2 (Liu et al., 2015) was used to exclude random connection. Finally, the degree centrality maps (weighted) were taken into statistical analysis. The difference image was obtained between before- and after-psilocin injection using voxel by voxel two-sample test; *p* < 0.05 was seen as statistically significant.

For ROI-wise functional connectivity analysis, in total 59 ROIs (Supplementary Table 1) were used according to the rat Sigma function brain atlas (Barrière et al., 2019). In the templates, the functional cingulate cortex was subdivided into Cg1, Cg2, and Cg3 (Supplementary Figure S1A). The average time courses of the 59 ROIs were extracted and the correlation strengths between each of them were calculated. Fisher-z transformation was performed on the correlation matrixes and the difference ROI-ROI pairs between before- and after-psilocin were acquired using paired-sample test. To display the significantly changed ROI-ROI pairs in ROI-wise FC analysis, a threshold of *p* < 0.05 was used for the statistical analysis.

For voxel-wise (seed-based) functional connectivity analysis, anatomical cingulate cortex (Supplementary Figure S1B) was chosen as the ROI and their average time course was extracted. All the correlation strengths between the time courses of voxels in the brain and the ROI time course were calculated. Fisher-z transformation was performed on the correlation maps and the difference images between before- and after-psilocin were acquired using voxel by voxel two-sample test; *p* < 0.05 was seen as statistically significant. To ensure reproducibility, a Supplementary Table S1 detailing the spatial correspondence between functional ROIs (SIGMA template) and anatomical ROIs (Paxinos atlas) is provided, with Cg1 and Cg2 mapped to the anterior cingulate cortex (ACC) and Cg3 to the medial cingulate cortex (MCC) in humans (Vogt and Paxinos, 2014).

## 3 Results

### 3.1 Psi treatment induced HTR bouts of rats

**Figure 1:**
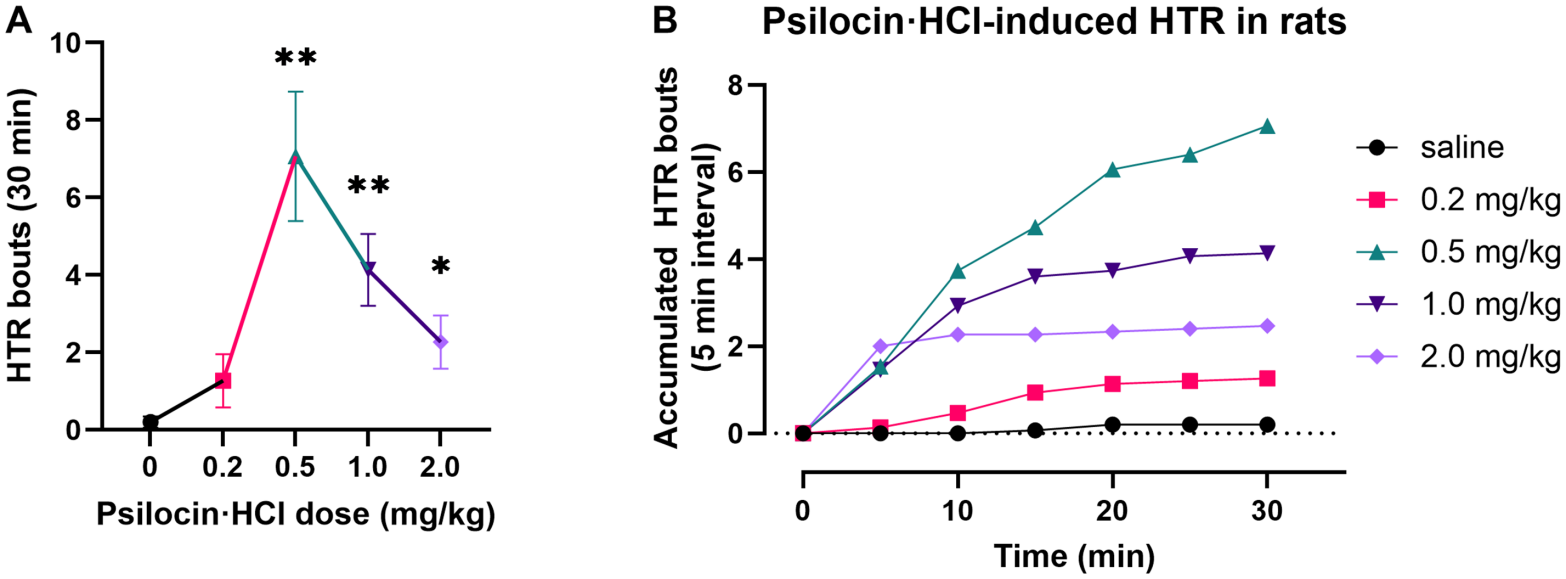
Psi increased HTR of rat. Dose–response **(A)** and time-response **(B)** of Psi induced HTR. Psi dose-dependently induced rapid HTR in rats and a dose of 2.0 mg/kg Psi reached the peak HTR effect within 5 min. Data are shown as mean ± SEM, one-way ANOVA followed by Dunnett’s multiple comparisons, *p < 0.05, ** *p* < 0.01, *n* = 15 per group.

Psi induced rat HTR bouts in a dose-dependent manner, presenting a characteristic bell-shaped dose–response curve (Figure 1). Low doses elicited minimal HTR bouts, while the intermediate dose (0.5 mg/kg) produced the peak response. In contrast, higher dose (1.0, 2.0 mg/kg) resulted in reduced HTR frequencies—though both the intermediate and high dose of Psi significantly increased rat HTR bouts relative to the saline group. The time-response curve revealed that HTR bouts primarily initiated 5–10 min after Psi injection and accumulated progressively over time.

Although 0.5 mg/kg Psi shows the most robust HTR effects, which primarily resulted from 5-HT_2A_R activation (Halberstadt et al., 2011), the dose of 2.0 mg/kg was selected for the subsequent fMRI and immunofluorescence experiments. This choice was motivated by psilocin’s polypharmacological profile: it acts on a diverse array of receptor systems, including multiple 5-HT subtypes, dopamine receptors, and other neurotransmitter targets (Tylš et al., 2014). And the dose of 2.0 mg/kg balances the 5-HT_2A_R engagement and may capture its broader neuropharmacological effects.

### 3.2 Mixed pattern of BOLD signals after acute psi treatment

**Figure 2:**
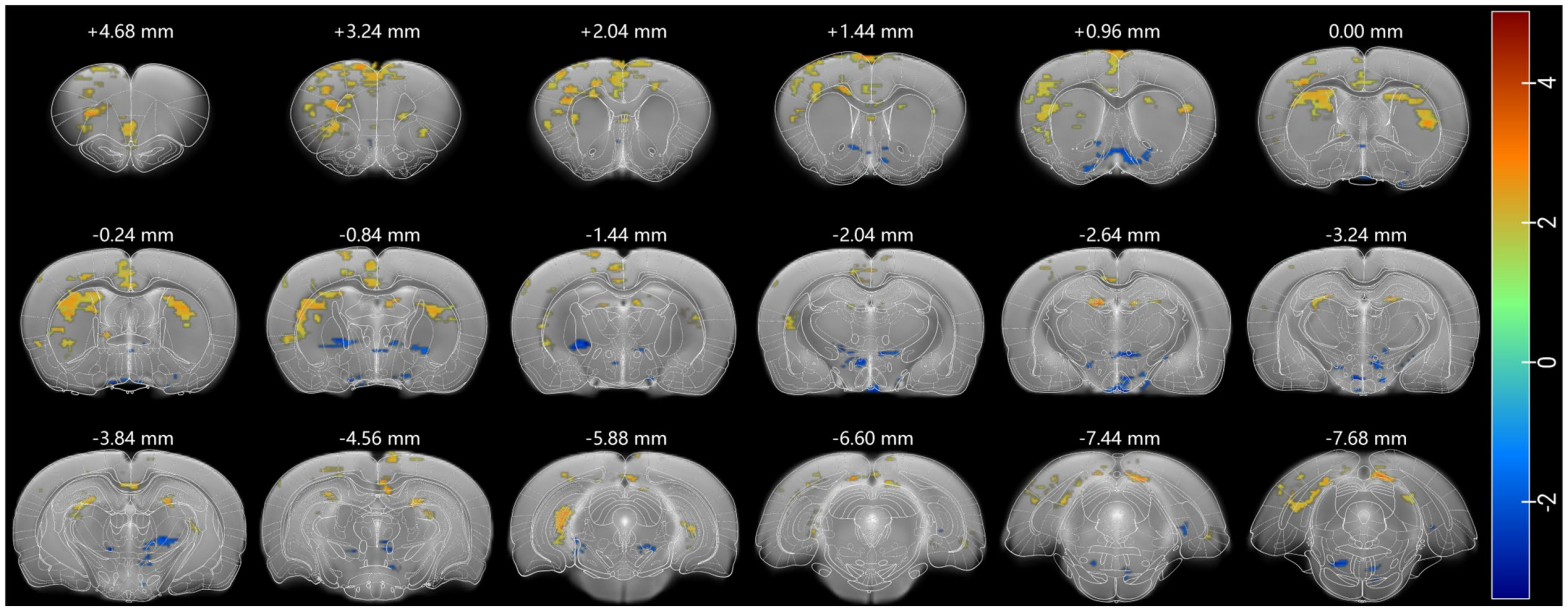
Psi produced a mixed pattern of degree centrality (DC). Increased DC (red) was widespread in cortical, striatal, and hippocampal regions and decreased in subcortical regions. A threshold of 0.2 was used to exclude random connection, DC was analyzed using two-sample test, and *p* < 0.05 was seen as statistically significant. Each positive (or negative) numerical value over brain sections is the AP value in the rat stereotaxic atlas, representing the distance anterior (or posterior) to bregma.

To investigate how psilocin affects rat brain activities, whole-brain resting state fMRI was performed and the degree centrality was analyzed before *versus* after a single dose of 2.0 mg/kg psilocin hydrochloride. The results revealed a mixed pattern of brain activity alteration (Figure 2), with increased degree centrality observed in the cingulate cortex, prefrontal cortex (PFC), motor cortex, somatosensory cortex, insular cortex, dorsal striatum, hippocampus, and the superior part of some septal and thalamic areas. Conversely, decreased degree centrality was noted in the ventral striatum, inferior part of septum, basal thalamic nuclei, and some hypothalamic areas. The psilocin-induced mixed pattern of brain activity changes in rats shows some similarity to the acute effects of its prodrug psilocybin in human (Preller et al., 2020).

### 3.3 Functional connectivity between cingulate cortex and other regions

**Figure 3:**
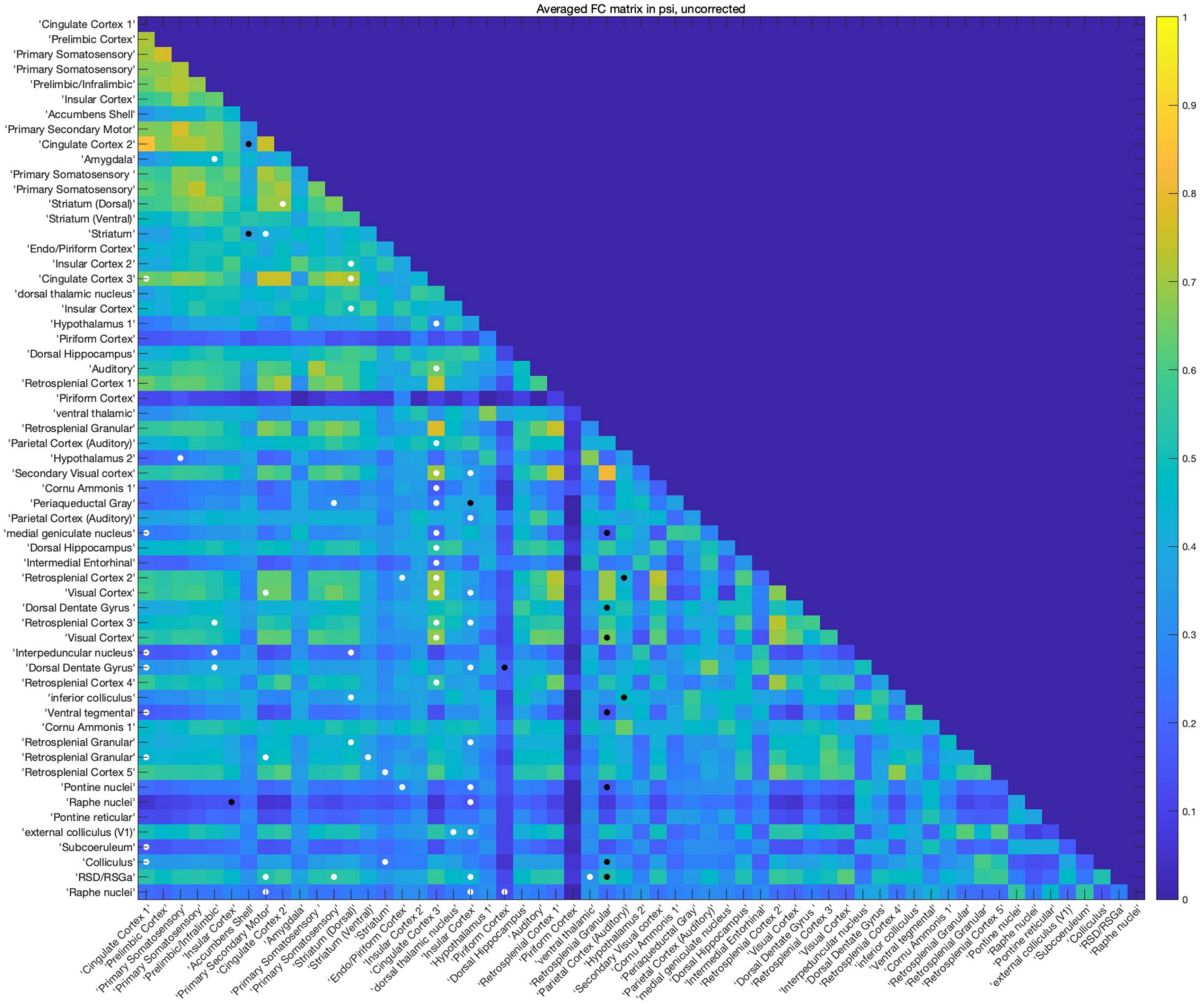
Psi changed brain functional connectivity. The rat brain was divided into 59 ROIs, FC between each ROI was analyzed using paired-sample *t* test without multiple correction, a threshold of *p* < 0.05 was used for the statistical analysis. White dots indicate increased FC between the two ROIs and black dots indicate decreased FC between them. Among the matrix, the FC of Cg1, Cg3, and striatum changed most.

We further explored the interconnection changes across the whole brain using ROI-wise FC analysis. The analysis matrix indicated that a substantial number of regions exhibited increased FC with multiple regions, including the cingulate cortex, dorsal and ventral striatum, prelimbic/infralimbic areas, piriform cortex, primary somatosensory cortex, motor cortex, and ventral thalamic nucleus. (Figure 3). Among these, the FC of the cingulate cortex underwent the most pronounced changes. Regions showing decreased FC with other areas included some of the insular cortex, accumbens shell, retrosplenial granular area, and parietal (auditory) cortex. And the FC of retrosplenial granular cortex showed the most significant reduction in FC. Additionally, bilateral fluctuations in FC changes were observed in some of the insular cortex and piriform cortex. Considering the possibility that multiple comparison correction might cover the potential positive results, no multiple comparison correction was performed in this exploratory analysis. Overall, the exploratory ROI-wise FC analysis suggested the FC of several rat cingulate subregions like the cingulate cortex 1 (Cg1), cingulate cortex 3 (Cg3), homologous to the respective ACC and MCC of humans and crucial for inter-hemispheric communication (Vogt and Paxinos, 2014) with other brain regions, exhibited substantial changes in FC with other brain regions.

### 3.4 Bidirectional FC changes of cingulate cortex with several cortical and subcortical regions

**Figure 4:**
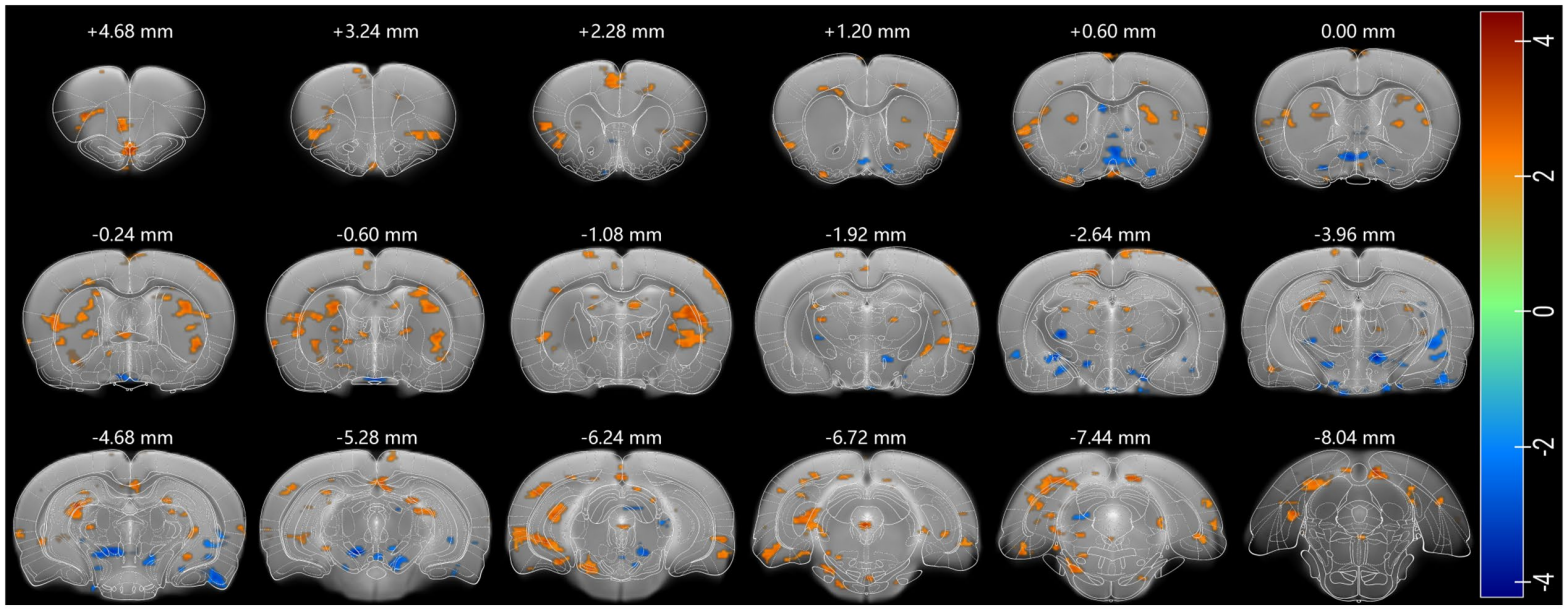
Psi-induced changes in Cg functional connectivity. Regions showing a positive coupling with Cg are shown in red, and negative in blue. Two-sample test was used and *p* < 0.05 was seen as statistically significant. Each positive (or negative) numerical value over the brain section is the AP value in the rat stereotaxic atlas, representing the distance anterior (or posterior) to bregma section.

To more precisely verify the key role of the cingulate cortex in psilocin-induced rat brain FC changes, seed-based FC analysis was performed using anatomical cingulate cortex (Paxinos and Watson, 2007) as the seed region (Supplementary Figure S1B). Psilocin was observed to increase rat cingulate connectivity with the olfactory bulb, orbital frontal cortex, anterior insular cortex, lateral part of somatosensory cortex, posterior entorhinal cortex, visual cortex, retrosplenial cortex, striatum, thalamus, hippocampus, postsubiculum, and periaqueductal grey, and decreased cingulate connectivity with the preoptic area, inferior part of septum, piriform cortex, amygdala, inferior thalamic areas, and substantia nigra (Figure 4).

### 3.5 Psi increased EGR1 expression throughout the rat brain

**Figure 5:**
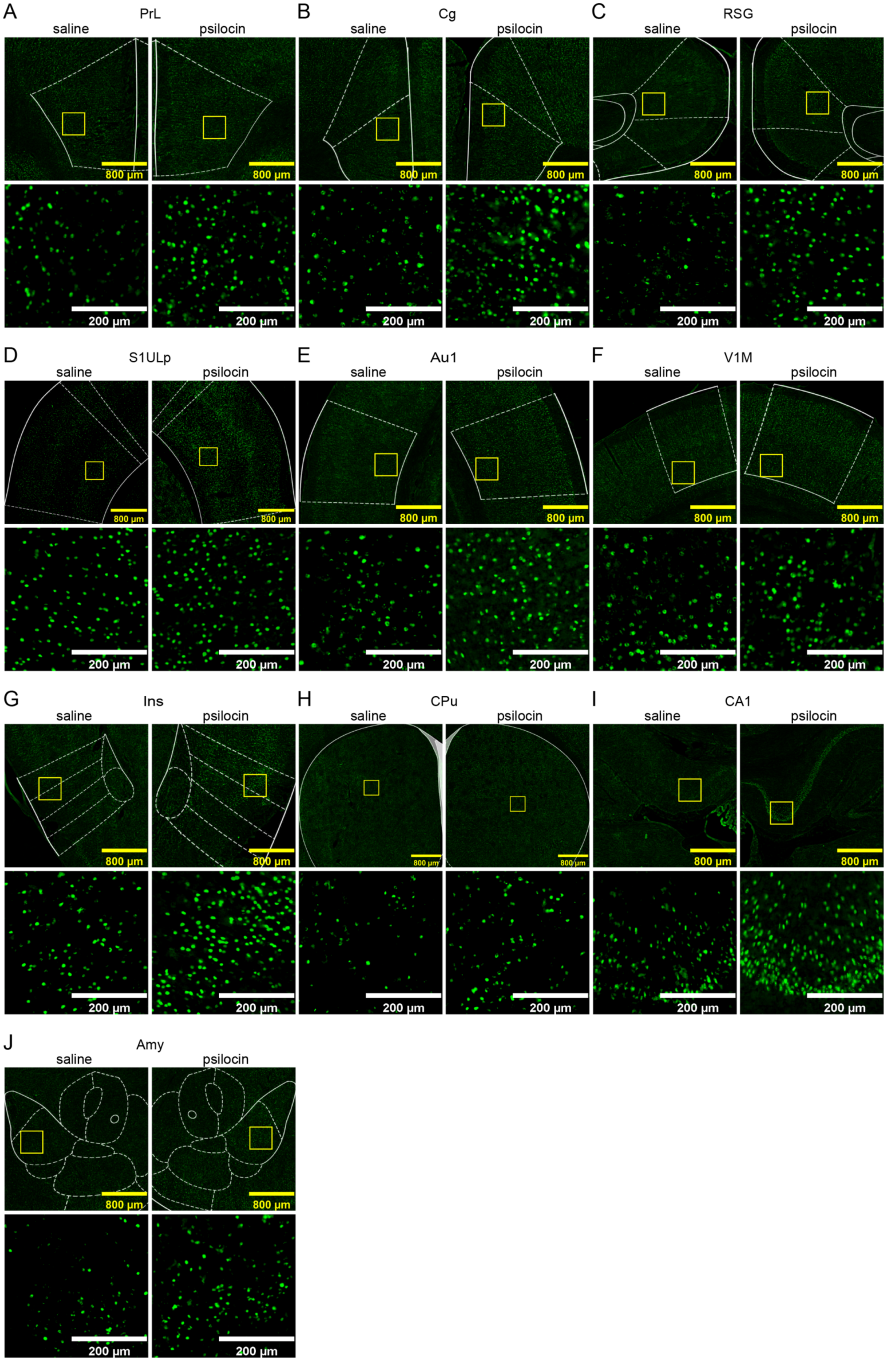
Representative immunofluorescence images of increased EGR1 in different regions **(A–J)**. **(A)** Prelimbic region, PrL; **(B)** cingulate cortex, Cg; **(C)** retrosplenial granular cortex, RSG; **(D)** primary somatosensory cortex, upper lip, S1ULp; **(E)** primary auditory cortex, Au1; **(F)** primary visual cortex, monocular, V1M; **(G)** insular cortex, Ins; **(H)** caudate putamen (striatum), CPu; **(I)** filed CA1 of the hippocampus, CA1; **(J)** amygdala, Amy. Scale bars, 800 μm in yellow, 200 μm in white.

**Figure 6:**
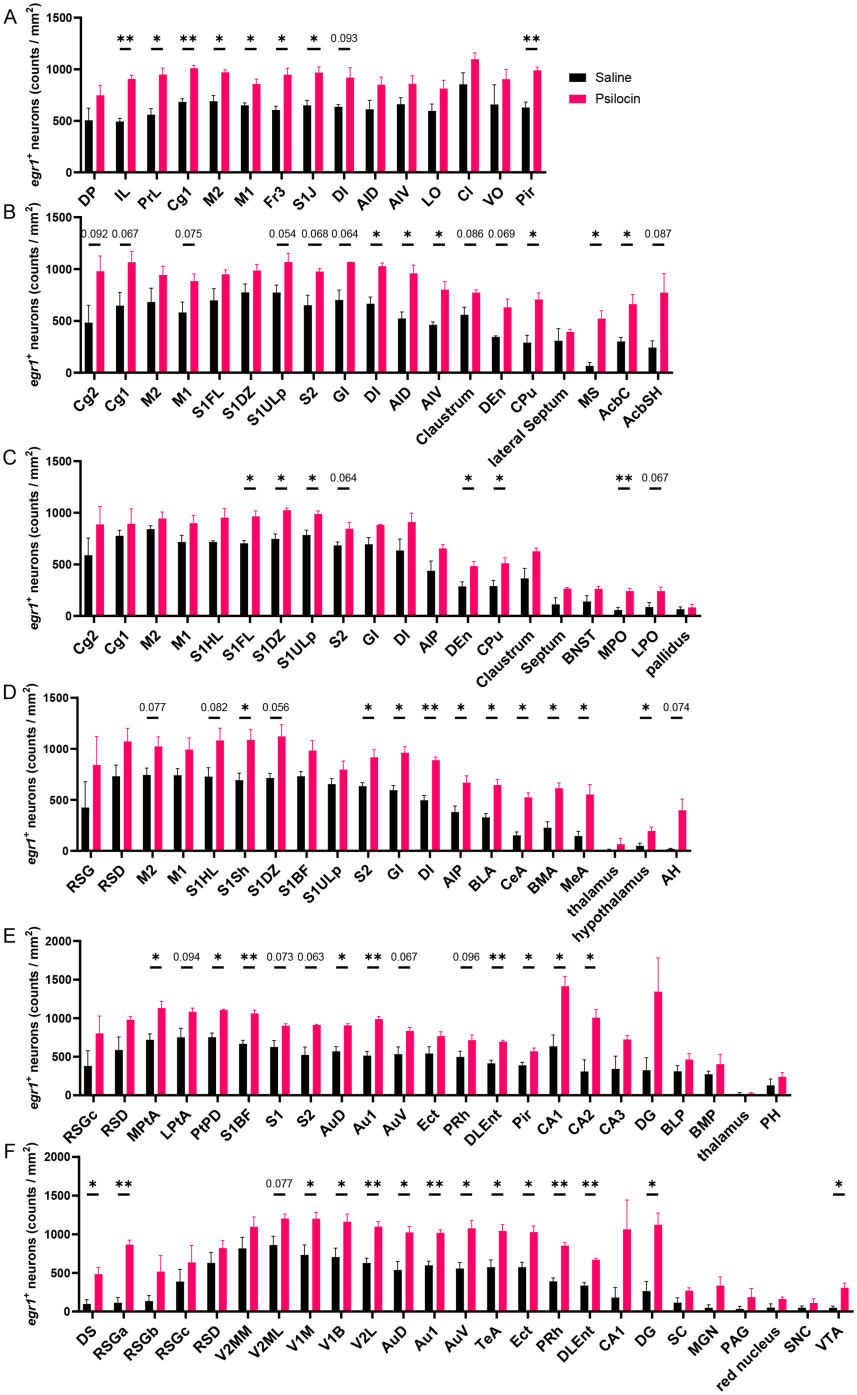
Increased EGR1 expression after Psi. Density of EGR1 positive neurons in different coronal sections (AP): **(A)** +3.24 mm, **(B)** +0.96 mm, **(C)** −0.24 mm, **(D)** −2.04 mm, **(E)** −3.84 mm, **(F)** −5.88 mm. The abbreviations of the IF brain regions were listed in Supplementary Table S2. Data are shown as mean ± SEM, multiple unpaired *t* test, compared with saline group, *p < 0.05, **p < 0.01, *p* value ranging between 0.05 and 0.1 are directly annotated in the graphs, *n* = 3 per group.

To validate psilocin-induced acute changes in brain, as revealed by fMRI, immunostaining of EGR1 was performed and six slices were selected according to the significant DC changes in cortical and subcortical regions like the cingulate cortex, prefrontal cortex, striatum, hippocampus, retrosplenial cortex, and some thalamic regions. Representative images of these brain regions are shown in Figure 5. In our IF counting procedures, subregions showing significant differences in EGR1 expression were elaborately counted and illustrated in the same graphs. Psilocin tended to increase EGR1 expression across the brain, with significant increases in the primary somatosensory cortex, insular cortex, striatum, nucleus accumbens, medial septum, preoptic area, amygdala, hypothalamus, parietal and temporal association cortex, auditory cortex, piriform cortex, hippocampus, visual cortex, dorsal subiculum, retrosplenial granular cortex, visual cortex, and ventral tegmental area (Figure 6). Furthermore, no decrease in EGR1 level was observed in our IF images.

## 4 Discussion

In this study, psilocin-induced brain activity changes were investigated using BOLD fMRI and EGR1 IF techniques. DC analysis, ROI-wise FC analysis, and cingulate cortex-based FC analysis revealed a mixed pattern of brain activity and connectivity changes, characterized by increased activities and FC in the cortical and striatal regions, and decreased activity in certain subcortical regions. These results highlight the pivotal role of the hub regions, such as the PFC and cingulate cortex, in psilocin-induced brain activity alterations, which may be the mechanism through which psilocin exerts potent psychoactive effects.

Increased degree centrality was observed in several cortical and subcortical regions, such as the prelimbic areas, cingulate cortex, striatum, and hippocampus (Figure 2). In cortical regions, EGR1 expression was also significantly increased in somatosensory, auditory, visual, and insular cortices (Figures 5, 6). The widespread activation suggested psilocin induces a hyperactive state in rat cortices. Consistently, a positron emission tomography (PET) study in healthy volunteers reported that acute psilocybin, the prodrug of psilocin, induced a hypermetabolic state in most cortical regions, as evidenced by strikingly increased in glucose metabolism in the frontal cortex (Vollenweider et al., 1997). And However, in contrast to the acute effects, another long-term study reported specifically decreased glutamate and N-acetylaspartate (NAA) levels in ACC of patients with major depressive 1 week after psilocin treatment (Doss et al., 2021). These two studies imply that psilocin may elicit different acute *versus* chronic effects, which deserves further investigation. Congruent with human studies, consistent results were observed in depression-phenotype susceptible mice that optogenetic stimulation of mPFC produced antidepressive effects and concurrently increased EGR1 and FOS expression (Covington et al., 2010), aligning with our observation of EGR1 upregulation in rat cingulate and PFC. However, psilocin’s clinical efficacy in humans involves additional factors not captured in rodent models, such as physiological structure, psychological support, effective does and repeated dosing. Future studies in non-human primates could help bridge this translational gap by validating cingulate-centered network changes in species with more human-like brain architecture.

Cingulate regions in humans and rodents exhibit both similarities and dissimilarities. In humans, the cingulate cortex is a significant hub region for association, cognitive flexibility, emotional processing, learning, and memory. In rats, this hub region is also important for inter-hemispheric communication. With reference to anatomical structures, the cingulate cortex in rats is composed of Cg1 and Cg2 (Supplementary Figure S1B) (Paxinos and Watson, 2007), homologous to ACC and the medial cingulate cortex (MCC) in humans, respectively (Vogt and Paxinos, 2014). And human posterior cingulate cortex (PCC) is cytoarchitecturally equivalent to rat retrosplenial cortex (RSC), which includes both retrosplenial granular and dysgranular cortices (RSG/RSD) (Lu et al., 2012; Vogt and Paxinos, 2014). However, in the fMRI templates (Barrière et al., 2019) we used, the rat cingulate cortex was functionally divided into three subregions, Cg1, Cg2 and Cg3 (Supplementary Figure S1A). Among these, Cg1 and Cg2 were identified as homologous to ACC, and Cg3 to MCC in humans (Vogt and Paxinos, 2014).

Our brain-wide FC analysis revealed mostly increased interconnection of Cg3 with other brain regions, and Cg1 and ventral striatum also exhibited significant changes in interconnection (Figure 3). Similarly, IF results showed that psilocin increased EGR1 in some cingulate regions (Figure 6). Given the importance of cingulate cortex in antidepressant-like effects of psilocin, cingulate cortex-based (Supplementary Figure S1B) seed analysis was performed using the anatomic cingulate cortex (Supplementary Figure S1B). This analysis showed that cingulate cortex exhibited hyperconnectivity with wide cortical and some subcortical areas including the olfactory bulb, orbital frontal cortex, insular cortex, entorhinal cortex, visual cortex, retrosplenial cortex, striatum and hippocampal dentate gyrus, and hypoconnectivity with septum, piriform cortex, amygdala, inferior thalamic areas, and substantia nigra (Figure 4). Congruent results were observed in human researches. Patients with major depressive disorder (MDD) show decreased module allegiance matrix (MAM) value between ACC and different regions like the insular cortex, amygdala, precuneus, and thalamus, suggesting a lowered interconnection strength in depressive state (Zheng et al., 2018), which was reversed after psilocybin administration, characterized by increased inter-regional FC, decreased brain modularity, and reduced hierarchical organization following the antidepressant treatment (Roseman et al., 2014; Carhart-Harris et al., 2017; Doss et al., 2021; Daws et al., 2022; Girn et al., 2022). These studies collectively imply the pivotal role of the cingulate cortex in psilocin-induced brain activity changes.

Although consistent fMRI results were observed, reproducibility remains a critical concern in brain imaging research-especially given that limited sample sizes (a key limitation noted for the studies included in our analysis) are well-documented to undermine the reliability of brain-behavior associations. As highlighted by recent large-scale reanalysis, small-sample fMRI studies often suffer from inflated effect sizes and low replicability, making it challenging to draw robust conclusions solely from neuroimaging evidence (Callaway, 2022; Marek et al., 2022). In line with these field-wide concerns, larger samples will need in the future research. Our present work emphasizes that while fMRI provides valuable insights into psilocin’s effects on brain connectivity, complementary methodologies, such as molecular biology assays, are essential to validate these findings and unravel the underlying biological mechanisms. The multi-pronged approach not only addresses the reproducibility gap but also strengthens the translational relevance of our observations. A recent psilocybin study may help elucidate, in this study, weighted phase-lag index (wPLI) was used to quantify connectivity, a method robust to volume conduction, providing independent validation of our fMRI-based functional connectivity findings, and confirming that psilocin/psilocybin-induced cortical network reorganization is not dependent on imaging modality (Silverstein et al., 2025).

To further consolidate the confidence in our studies, immunofluorescence of EGR1, a well-recognized neural activation marker, was performed. Elevated EGR1 expression in both cortical and subcortical areas revealed a brain-wide ‘neural activation’ blueprint, although in some regions, increase in EGR1 was only a trend but did not reach statistical significance. The two experiments showed convergent neural activation in many brain regions including the prefrontal, cingulate, sensory-related, insular, and retrosplenial cortex, hippocampus, and striatum, which are closely related to sensory processing, emotion, association, cognitive flexibility, and memory. Notably, EGR1’s widespread upregulation (Figures 5, 6) aligns with recent evidence that psilocin triggers activity-dependent synaptic rewiring in frontal cortical networks (Jiang et al., 2025). EGR1 is a key mediator of synaptic plasticity, regulating the expression of genes involved in dendritic spine formation and synaptic strength (Duclot and Kabbaj, 2017). Our finding of EGR1 elevation in cingulate, striatum, and hippocampus—regions critical for mood and cognition—suggests that psilocin may promote cortical plasticity via EGR1-dependent pathways which deserve further exploration. Collectively, our data link psilocin’s network-level effects (fMRI) to molecular mechanisms of plasticity (EGR1) and structural remodeling (synaptic rewiring), forming a coherent “network-plasticity-molecule” pathway.

The integrative brain dark energy model (Gong and Zuo, 2025) and two electrophysiological studies (Thomas et al., 2022; Silverstein et al., 2025) may help further elucidate our results. According to brain “dark energy” theory, the intrinsic energy consumption supports spontaneous neural oscillations (SSOs) that maintain the brain’s intrinsic functional architecture-accounting for ~70% of total cerebral energy expenditure. The integrative model was proposed that spontaneous neural oscillations (SSOs), organized into a hierarchical frequency architecture (slow-1 to slow-6) via the natural logarithm linear law (N3L), are the core carriers of brain dark energy (Gong and Zuo, 2025). Our findings on psilocin-induced brain changes provide critical *in vivo* evidence for the drug modulation of brain dark energy, aligning closely with this hierarchical SSO framework.

Silverstein et al. (2025) used high-density EEG in rats to show that psilocybin dose-dependently disrupts theta-gamma phase-amplitude coupling, while increasing frontal high gamma connectivity and posterior theta network density. These oscillatory changes align with our fMRI observations of enhanced cingulate-striatal/cortical connectivity (Figures 3, 4): theta oscillations correspond to SSOs’ slow-4 band (sensorimotor/cognitive processing) and gamma to slow-3 band (local integration), both core “computation level” components of dark energy (Gong and Zuo, 2025). The theta-gamma decoupling further explains our finding of mixed activity changes-psilocin reallocates dark energy from local theta-modulated processing to long-range gamma-mediated integration. And EGR1 IF validated widespread cortical/subcortical activation (Figures 5, 6), implicating that psilocin may target SSO-related neural activity rather than task-evoked responses.

Thomas et al. (2022) complemented this by demonstrating that psilocin acutely disrupts mouse sleep–wake architecture (delayed REM onset, fragmented NREM sleep) and enhances 3–5 Hz oscillations in mPFC LFP. This 3–5 Hz rhythm matches SSOs’ slow-4/5 bands (modulation level, long-range integration) and correlates with quiet wakefulness—supporting our fMRI result of cingulate cortex hyperconnectivity (a key slow-5 hub). Notably, psilocin slowed the recovery of sleep slow-wave activity in mPFC LFP, indicating localized modulation of SSO homeostasis that parallels our fMRI-derived network changes.

Collectively, these electrophysiological findings suggest our fMRI results: psilocin targets frequency-specific SSOs (slow-3/4/5), disrupting oscillatory coupling and reorganizing network connectivity to reallocate dark energy. This cross-scale convergence (macroscopic fMRI networks ↔ microscopic oscillations) reinforces the hypothesis that psilocybin/psilocin exerts potential therapeutic effects by normalizing SSO dysregulation in mood/cognition-related bands. Future studies could integrate multi-band fMRI SSO analysis with EEG/LFP to directly map psilocin’s effects to specific slow bands, clarifying dark energy modulation at the network-oscillation interface.

Despite the overall coherence, there were still undesirable but predictable discrepancies between fMRI and IF results necessitating detailed elucidation. For example, regions with positive BOLD signals are not always correspond to those with significantly increased EGR1 level. In addition, decreased BOLD signals were observed in some thalamic and hypothalamic regions, whereas no reduction in EGR1 level was observed.

Understanding the principle of BOLD fMRI is crucial to interpreting the inconsistencies. All brain activities are energy-consumptive, including both excitatory and inhibitory processes. BOLD signals indirectly reflect the mass action of neuronal assemblies through hemodynamic changes induced by varied brain processes (Glover, 2011), known as neurovascular coupling, which is ambiguous in its nature (Buzsáki et al., 2007). In substantial cases, excitation consumes more energy than inhibition (Buzsáki et al., 2007). However, the relationships between BOLD signals and neural activities are still not fully understood, especially when interpreting negative signals. From a perspective of excitation-inhibition balance, a positive fMRI signal in a brain region is usually associated with increased activity, which may result from (1) direct excitation activation, (2) proportional activation of both excitation and inhibition leading to unchanged local neural activity but increased metabolism, or (3) indirect excitation activation secondary to deactivation of inhibition, which causes net excitation in the conditions. However, given the complexity of hierarchical organization and multiplicity of circuits and microcircuits, net inhibition in a certain brain region may generate either positive or negative BOLD signals in a circuit-dependent manner (Logothetis, 2008). For example, early shunting of an excitatory input may lead to decreased recurrent excitation, in which a clear negative BOLD signal occurs, while increased synaptic inhibition or early shunting of an output may result in a positive signal (see the review for detail (Logothetis, 2008)).

It has been reported that isoflurane enhanced inhibition of thalamic neurons in the ventrobasal thalamus via GABA_A_R-dependent mechanisms, but in reticular thalamic nucleus (RTN) via GABA_A_R-independent mechanisms (Ying et al., 2009), which may help to interpret the negative BOLD signals in basal thalamic nuclei in our fMRI readouts.

Apart from GABAergic inhibition enhancement (Fracasso et al., 2022), activation of dopamine system can also elicit decreased striatal fMRI signals in rats via dopamine D_2_ receptor (D_2_R) stimulation (Shih et al., 2009; Hsu et al., 2014). A human PET study has shown that psilocybin indirectly facilitates dopamine release through stimulation of 5-HT_1A_ and 5-HT_2A_ receptors (Vollenweider et al., 1999). Furthermore, psilocybin has been shown to increase FC within 5-HT-associated networks while decreasing FC within dopamine-associated networks in mice (Grandjean et al., 2021). Given that our IF results demonstrated psilocin elevated EGR1 level in dopamine-related regions such as ventral tegmental area (VTA) (Figure 6), we therefore speculate that psilocin-induced negative subcortical BOLD signal may be attributed to dopamine or GABA activation or a combination of both.

A ‘bipartite’ model theory has been proposed that brain serotonin mediates adaptive responses to adversity through 5-HT_1A_R signaling for stress modulation and 5-HT_2A_R signaling for adaptation, respectively (Carhart-Harris and Nutt, 2017). In our study, the psilocin-induced hyperactivity across cortical and some subcortical regions closely matched the distribution of 5-HT_2A_ receptors. This finding is consistent with the spatially heterogeneous gene expression pattern of 5-HT_1A_ and 5-HT_2A_ Receptors across primate and rodent brains (Burt et al., 2018; Preller et al., 2020; Grandjean et al., 2021), indicating the crucial role of the two receptors in psilocin-induced brain activity alteration.

BOLD fMRI scanning detects the neurovascular coupling event, and stimulation-induced cerebral blood flow (CBF) is an important factor worth considering. Brain 5-HT acts as a major vasoconstrictor, and increases in 5-HT levels, both directly and indirectly, leads to reduced CBF (Cohen et al., 1996). Several serotonin receptors are involved in the complicated vasculature effects, including 5-HT_1B_, 5-HT_1D_, and 5-HT_2A_ receptors, and psilocin has high affinity for these receptors, making the BOLD signals challenging to interpret (Cohen et al., 1996; Elhusseiny and Hamel, 2001; Hamel, 2006; Lewis et al., 2017). However, a hemodynamic study in humans suggests that psilocybin decreases global CBF but bidirectionally modulates regional CBF, with increases in the right frontal and temporal regions, bilateral fluctuation in the anterior insula, and decreases in the left parietal and occipital regions, amygdala, globus pallidus, insula, and thalamus, as measured using pseudo continuous arterial spin labeling (pCASL) fMRI (Lewis et al., 2017). This pattern indirectly reflects a hyper-frontal and hypo-subcortical state, corresponding to our fMRI readouts to some extent.

Given that anesthesia can influence neural activity, our experimental design, which involved performing BOLD fMRI and EGR1 IF simultaneously but using separate rats from the same batch, was based on the following considerations.

Serotonergic neurons in the dorsal raphe nucleus (DRN) were reported to be inhibited following isoflurane anesthesia (Johansen et al., 2015; Yang et al., 2019), which could influence the activities of brain-wide neurons expressing serotonin receptors (Mukaida et al., 2007) and even reconfigure the prefrontal neurotransmitter network composing of glutamate, GABA, dopamine, acetylcholine, adenosine, norepinephrine, histamine, and serotonin (Zhang et al., 2020). However, considering the potential influence of mental stress induced by forced fixation and the common HTR effect (Figure 1) produced by hallucinogenic 5-HT_2A_ receptor agonists in awake rodents, fMRI scanning under an anesthetic state is an eclectic but necessary method for rodent neuroimaging.

General anesthesia prevents activity-induced initiation of IEG *egr1* transcription, consequently resulting in reduced EGR1 level, but does not stop ongoing *egr1* transcription or influence EGR1expression (Bunting et al., 2015). Importantly, isoflurane-elicited prolonged changes of protein expression in rats could persist for several days (Kalenka et al., 2010). Therefore, to avoid blunting psilocin-induced EGR1 changes under post-isoflurane conditions, IF rats were kept unanesthetized until the transcardial perfusion operation. In addition, IEGs are critical mediators of gene × environment interactions (Guzowski et al., 1999), so EGR1 IF in this study may reflect the combined effects of psilocin and potentially environmental stimuli, which may contribute to additional differences between BOLD signals and IF results. Experiments with conscious animals can better reflect the real conditions. Each rat was well acclimatized to the experimental environments and efforts were made to minimize the confounding influence of any environmental factors during the procedure.

Although this experimental design precluded one-to-one comparison between fMRI readouts and IF EGR1 counts for each rat and may introduce additional factors (anesthetic state or environment), the group-wide comparison still differentiated the affected brain regions as mentioned above.

Aisling Spain and her colleagues reported seemingly discrepant results, that rats with a single dose of 2 mg/kg psilocin exhibited a negative signal in the cingulate and somatosensory cortex, but a positive signal in the hypothalamus and amygdala (Spain et al., 2015). Here, we try to provide an explanation for these differences. In their research, a dose of 2 mg/kg psilocin was intravenously injected into rats, which may have led to a sharp rise in blood psilocin concentration. In contrast, in our study, 2.0 mg/kg psilocin hydrochloride was intraperitoneally administered through a homemade injection device, which likely maintained a milder and more sustained concentration, resulting in more moderate effects. Additionally, psilocin hydrochloride has a larger molecular mass than psilocin, suggesting that the effective dosage in our study was lower than that in Spain et al. (2015).

Our HTR results presented a bell-shaped curve in a dose-dependent manner, with the 2.0 mg/kg Psi located on the descending phase, still significantly increasing HTR bouts (Figure 1) suggesting different receptors may be involved in different phases. Psilocin has affinities for multiple serotonin receptors, including 5-HT_1A_ and 5-HT_2A_ receptors, which may constitute a “bipartite” model for serotonergic modulation, corresponding to passive and active coping, respectively (Carhart-Harris and Nutt, 2017). The inhibitory role of 5-HT_1A_ receptor in HTR has been reported (Shahar et al., 2022); and thus the descending phase may be due to the inhibitory effects of 5-HT_1A_ receptor activation (Darmani et al., 1990). The 2 mg/kg psilocin in their research may have resulted in stronger inhibitory effects. Given this, we speculate bioactive psilocin may exert biphasic effects on fMRI signals in a dose-dependent manner, which could account for the differences between the two studies. Furthermore, in Aisling Spain’s research, surgeries and stimulant paradigms were employed, which may have influenced the states of rats and introduced additional factors.

There are some technical considerations and limitations warranting further explorations. Brain activity and connectivity are almost bidirectional, characterized by simultaneously increased and decreased BOLD signals detected in varied brain regions, while only unilaterally increased EGR1 expressions, though not significantly in some regions, were observed. Furthermore, EGR1 is widely expressed cross-brain and the immunostained EGR1 is the cumulative expression of all cell types in certain regions, but whether it has a cell-type specific expressing pattern still remains unclear, which may complicate our understanding of excitation or inhibition of specific regions where the constituent neuron types are varied. In future research, bilaterally modulated biomarkers or a combination of different cell-type specific ones may help to understand the bidirectional fMRI signals and further elucidate specific cell types involved in these neural activities.

Activation of 5-HT_1A_ and 5-HT_2C_ receptors endows serotonergic system with an arousal-promoting effect, whereas receptors like 5-HT_1B_, 5-HT_2A_, 5-HT_3_, 5-HT_6_ and 5-HT_7_ receptors have little effect (Li et al., 2021). How psilocin influences the depth of anesthesia remains unclear. Psychedelics targeting 5-HT_2A_ receptors induce HTR in both mice and rats (Halberstadt et al., 2020), circumventing the fMRI scanning under awake condition. There may be a critical conscious-anesthetic state where HTR is totally inhibited and the brain activities closely resemble those in conscious state, which would be of particular significance for fMRI research into psychedelics in rodents.

In this fMRI study, predefined referential templates (Barrière et al., 2019) were used to parcellate the fMRI data, different to the stereotaxic atlas used in IF experiments (Paxinos and Watson, 2007). Mismatch or localization errors may occur, especially at the edges and in certain small nuclei, due to architectural and volumetric differences between individuals. Additionally, the margins of BOLD signal clusters may also vary, depending on the methodologies adopted (Guzowski et al., 1999). Therefore, higher resolution fMRI may be important for more accurate localization of affected brain regions (Romanov et al., 2019), and more mutually-matched atlases are needed for comparison.

Measurement reliability is also a fundamental prerequisite for reproducible findings and valid interpretation (Zuo et al., 2019). Our fMRI data were acquired using a 7 T Bruker system with a standardized resting-state protocol (GE-EPI sequence, TR = 2,000 ms, TE = 14 ms) and processed via rigorous pipelines (afni, fsl, ANTs) including motion correction, band-pass filtering, and spatial normalization to the SIGMA rat brain template (Barrière et al., 2019). As emphasized by Zuo et al. (2019), fMRI reliability strongly depends on scan duration and preprocessing rigor. Each rat underwent 10 min of scanning before and after psilocin administration (total 20 min of usable data), aligning with the minimum duration (20–30 min) recommended for adequate functional connectivity (FC) test–retest reliability in rodents (Elliott et al., 2019). But longer duration is need in future research. Head fixation, general anesthesia and standardized preprocessing mitigated artefactual variability, while convergent results from ROI-wise and seed-based FC analyses further support internal consistency. However, the relatively shorter scan time compared to human studies may limit FC reliability in subcortical regions with weaker BOLD signals (Zuo et al., 2019).

EGR1’s reliability is supported by evolutionary conservation and consistent induction by neuronal activity (González-Maeso et al., 2007; Pérez-Cadahía et al., 2011; Leal et al., 2014; Minatohara et al., 2016; Chandra and Lobo, 2017; Duclot and Kabbaj, 2017; Jefsen et al., 2021). Previous rodent studies demonstrate high consistency for EGR1 expression in cortical and striatal regions (González-Maeso et al., 2007; Jefsen et al., 2021). Our standardized IF protocol (4% PFA perfusion, 1:1,000 primary antibody dilution) and analysis with reference to the rat brain atlas (Paxinos and Watson, 2007) ensured consistency. Convergent activation patterns between EGR1 IF and fMRI (e.g., cingulate cortex, striatum, hippocampus) may further validate reliability.

## 5 Conclusion

Despite technical limitations, our findings demonstrate that psilocin induces hyperactivity and hyperconnectivity across various cortical regions, particularly in the cingulate cortex. This underscores the pivotal role of the ‘hub regions’ such as cingulate cortex, in mediating psilocin-induced brain activities and connectivity changes. Moreover, our integration of BOLD fMRI and EGR1 IF provides a novel approach to better understand the intricate effects of psilocin on brain function.

## Data Availability

The original contributions presented in the study are included in the article/Supplementary material, further inquiries can be directed to the corresponding authors.

## Footnotes

1
https://afni.nimh.nih.gov

2
https://fsl.fmrib.ox.ac.uk/fsl/fslwiki/

3
https://github.com/ANTsX/ANTs

4
https://people.cas.sc.edu/rorden/mricro/Bru2anz
