# Supplementary material for: Acute psilocin increased cortical activity in rat
Source: Front Neurosci. 2026 Feb 4;20:1593703. doi: 10.3389/fnins.2026.1593703 (PMC12915336; doi:10.3389/fnins.2026.1593703)
Supplement: Supplementary file 1 [file Data_Sheet_1.docx]

Supplementary Material

Acute psilocin increased cortical activities in rat

**Junhong Liu^1^, Yuanyuan Wang^1,2^, Ke Xia^1^, Jinfeng Wu^3^, Danhao Zheng^3^, Aoling Cai^4*^, Haitao Yan^1*^, Ruibin Su^1*^**

*** Correspondence:**

Aoling Cai
525420282@qq.com
Haitao Yan
yanht7809@aliyun.com
Ruibin Su
ruibinsu@126.com

**
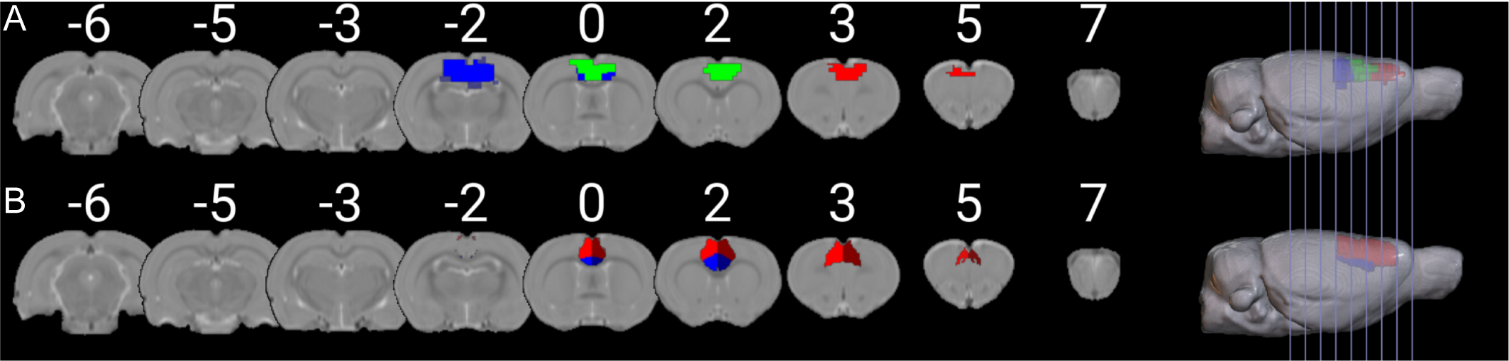
**

**Supplementary Figure 1.** (A) Functional and (B) anatomical coordinate of cingulate cortex. (A) red, green and blue represent the functional position of Cg1, Cg2 and Cg3, respectively in the rat Sigma function brain atlas(Barrière et al., 2019). (B) Red and blue regions represent the respective anatomical position of Cg1 and Cg2 according to the stereotaxic rat atlas(Paxinos and Watson, 2007).
